# Characteristics of Patient Complaints in a Large Tertiary Hospital in China: A Longitudinal Analysis from 2022 to 2024

**DOI:** 10.3390/healthcare14172745

**Published:** 2026-08-28

**Authors:** Baoxiang Wang, Xuyuan Kuang

**Affiliations:** 1 Department of Health Management, Xiangya Hospital, Central South University, Xiangya Rd 87, Changsha 410008, China; 2 Department of Hyperbaric Oxygen, Xiangya Hospital, Central South University, Xiangya Rd 87, Changsha 410008, China; 3 Department of Hyperbaric Oxygen, Xiangya Jiangxi Hospital, Central South University, Fenghe North Avenue 266, Nanchang 330006, China

**Keywords:** patient complaints, healthcare quality, general hospital, patient experience, trend analysis

## Abstract

Background: Patient complaints provide critical insights into healthcare quality and patient experience, serving as a direct feedback mechanism for service improvement. Objective: This study aimed to characterize major complaint categories, departmental distribution, personnel attribution, and resolution effectiveness of patient complaints at a large Chinese tertiary hospital using empirical data from 2022 to 2024. Methods: A retrospective observational design was conducted on complaint records (2022: *n* = 187; 2023: *n* = 192; 2024: *n* = 201) at Xiangya Hospital. All data presented are empirically observed records from the hospital’s internal complaint management system; no hypothetical projections or modelled data are included. Complaints were independently categorized into six themes by two researchers (Cohen’s κ = 0.84). Resolution effectiveness was coded by the hospital’s complaints office. Descriptive statistics and chi-square tests were performed using R software. A complementary SWOT analysis was conducted to assess institutional complaint management capacity. Results: Service attitude (25.1% in 2022, 23.4% in 2023, 21.9% in 2024) and communication (20.3%, 18.8%, 17.2%) constituted the largest complaint categories, both showing significant declining trends (χ^2^_trend = 6.54, df = 2, *p* = 0.038 for communication; χ^2^_trend = 5.98, df = 2, *p* = 0.045 for service attitude). The top three departments with the highest complaint volumes were General Surgery (16.6% in 2022), Gastroenterology (12.8%), and Otolaryngology (9.6%). Seasonal analysis revealed that the third quarter (July–September) was the peak complaint period (29.4% in 2022). The overall effective resolution rate improved from 62.3% in 2022 to 67.7% in 2023 and 71.5% in 2024 (χ^2^_trend = 8.12, df = 2, *p* = 0.017). Treatment outcome complaints showed a relative increase from 18.2% in 2022 to 20.1% in 2024, while billing/administrative complaints increased from 15.0% to 17.4%. Conclusion: Systematic analysis of patient complaints effectively identifies recurrent issues and informs quality improvement strategies. A balanced focus on both “soft skills” (e.g., communication, empathy) and “hard system” factors (e.g., processes, resources) is essential. Continuous monitoring and responsive feedback mechanisms are crucial for sustaining improvements in patient safety and satisfaction.

## 1. Introduction

The continuous improvement of modern healthcare systems and the increasing awareness of patients’ rights have made patient feedback a crucial metric for assessing the quality of healthcare services, patient satisfaction, and the overall harmony of doctor–patient relationships. As healthcare institutions strive to enhance service quality and promote patient-centred care, analysing patient feedback has emerged as an essential tool for identifying deficiencies, guiding improvements, and informing strategic decision-making in healthcare management and clinical practices [1,2].

While traditional patient complaints are expressions of dissatisfaction, modern feedback mechanisms increasingly capture a broader range of patient concerns. These include not only formal complaints but also consultation requests, reflecting patients’ desire for clarification, guidance, and assistance with the healthcare system [3]. This shift indicates growing patient engagement and a preference for proactive problem-solving rather than adversarial confrontation. Patient complaints together offer valuable insights into gaps in healthcare delivery. They highlight issues related to diagnostic accuracy, treatment effectiveness, communication skills, service accessibility, and administrative efficiency and may uncover systemic challenges that remain hidden in conventional satisfaction surveys [4,5]. Effective handling and analysis of complaints are associated with better patient safety, higher satisfaction, and improved clinical outcomes [1]. Furthermore, longitudinal analysis of such data enables institutions to detect emerging patterns and adopt timely, targeted interventions [6].

Systematic reviews of the medical complaint literature have identified three major domains of complaint triggers: clinical treatment (diagnostic accuracy, treatment efficacy, safety), hospital administration (waiting times, accessibility, costs), and physician–patient relationship (communication quality, empathy, respect) [7]. These domains are not mutually exclusive; rather, they interact to shape patients’ overall care experiences. Notably, recent reviews have highlighted that complaint management itself is an understudied area, with key challenges including delayed responses, passive handling attitudes, inadequate accessibility for vulnerable populations, and a lack of effective monitoring and feedback mechanisms [7]. This study addresses these gaps by providing a longitudinal, multi-dimensional analysis of complaint patterns and resolution effectiveness in a large Chinese tertiary hospital.

In rapidly developing healthcare systems, formal channels have been established to collect and address patient feedback. In China, the “12345” mayor’s hotline, an official, government-operated public service platform, plays a key role in gathering and forwarding public grievances, suggestions, and service inquiries to relevant institutions [8]. In the healthcare context, this hotline captures a wide range of patient-initiated interactions. The volume and nature of work orders processed through this channel reflect patients’ satisfaction, expectations, and informational needs, offering an opportunity to improve institutional responsiveness.

Research has shown that different types of patient feedback correspond to various domains of healthcare delivery, such as diagnostic accuracy, procedural execution, nursing care, communication, cost transparency, and administrative logistics [2,4,9]. For instance, complaints related to medical quality often involve issues such as diagnostic errors, inadequate treatment efficacy, or safety breaches [5,9]. Communication-related complaints typically reflect deficiencies in information exchange, poor service attitudes, or insufficient explanation of medical procedures [10]. Meanwhile, feedback concerning hospital management often centres on physical infrastructure, wait times, and billing discrepancies [4,9]. The seasonal distribution of feedback can further reveal systemic vulnerabilities; for instance, peaks during high-demand periods like summer or flu season may signal pressure points in staff allocation, communication workflows, and patient throughput [11,12]. Analysing these temporal patterns enables proactive improvements in service delivery and patient support during peak seasons.

Nationwide surveys have further revealed that complaint management capacity varies substantially across institutional levels. Guo et al. [13] found that while 97.1% of tertiary hospitals had dedicated complaint departments, this proportion dropped to 88.9% in primary hospitals and 82.8% in ungraded facilities. Similarly, routine complaint data analysis for service improvement was conducted in 87.0% of tertiary hospitals but only 42.0% of primary hospitals. These disparities underscore the need for context-specific complaint management strategies and highlight the value of examining complaint patterns within well-resourced tertiary settings as benchmarks for broader system improvement.

While previous studies have proposed various management frameworks for complaint handling—ranging from standardized complaint taxonomies [1,12] to SWOT (Strengths, Weaknesses, Opportunities, Threats) situational analysis [14]—the integration of complaint pattern analysis with institutional capacity assessment remains limited. This study contributes to this gap by complementing complaint trend analysis with a structured SWOT assessment of the hospital’s complaint management system, drawing on the methodological approach validated in oncology hospital settings [14].

This study aims to systematically analyse patient complaints received via the hospital’s internal complaint management system at a large tertiary hospital in Central China from 2022 to 2024. By examining trends in volume, complaint types, departmental and personnel attribution, seasonal fluctuations, and resolution effectiveness, this study provides evidence to guide targeted interventions in hospital operations, communication strategies, and quality improvement systems. The specific research questions are as follows: (1) What are the main categories and departmental distributions of patient complaints? (2) How do complaint patterns change over time and across seasons? (3) What is the effectiveness of complaint resolution, and how can it be improved?

## 2. Methods

### 2.1. Study Design and Data Sources

This study adopted a retrospective observational design, analysing patient complaint data collected through the hospital’s centralized patient feedback and complaint management system from 1 January 2022 to 31 December 2024 at Xiangya Hospital, a major academic tertiary hospital in Central China. A complaint was defined as a patient or family member expressing dissatisfaction and requesting improvement or resolution. All complaint work orders were extracted from the hospital’s internal patient feedback system and compiled using Microsoft Excel. The data were reviewed and validated by trained hospital administrative staff to ensure accuracy and completeness. All work orders received during the study period were included, with no exclusions applied. All data presented in this study (2022, 2023, and 2024) are empirically observed records from the hospital’s internal complaint management system. No hypothetical projections or modelled data are included in the results.

Data source contextualization: Unlike studies relying on government hotline platforms [3], medico-legal dispute compensation cases [15], or provincial survey data [13], our data were derived from the hospital’s internal complaint management system, capturing the full spectrum of grievances—from minor service attitude concerns to major clinical disputes. This approach enables the identification of “subclinical” complaints that may not escalate to compensation or litigation, thereby providing a more comprehensive picture of patient experience gaps.

Hospital activity-related data are as follows: In 2022, the hospital recorded approximately 1.85 million outpatient visits, 82,000 hospital discharges, and 45,000 surgical procedures.

### 2.2. Aims and Objectives

The aim of this study was to systematically analyse patient complaints at a large tertiary hospital in Central China. The study evaluated the distribution of complaint categories, departmental and personnel attribution, seasonal patterns, and resolution effectiveness in 2022 and projected changes in complaint composition and resolution rates through 2024 following targeted interventions. By integrating these analyses, the study sought to generate actionable insights to strengthen hospital management, enhance communication strategies, and improve the quality, responsiveness, and patient-centredness of healthcare services.

#### 2.2.1. Complaint Categorization Framework

To enable standardized analysis, all complaint cases were categorized using a dual-dimensional classification system based on national hospital complaint handling guidelines and validated models from the literature [1]. Our six-category framework was developed by synthesizing validated taxonomies from the literature. Reader et al. [1] proposed a three-domain classification (clinical, management, and relationship), which has been widely adopted [7,12]. Subsequent studies have refined this taxonomy to include specific subcategories such as service attitude, communication, billing, and waiting times to better capture context-specific complaint drivers [3,14,15]. Drawing on these established frameworks and our hospital’s complaint records, we adopted six categories that balance analytical granularity with coding reliability. Our classification framework focuses on the root cause categories of complaints (e.g., service attitude, communication) rather than specific clinical incident types (e.g., postoperative infection, medication error). This design choice, consistent with the classification logic of Guo et al. [13], facilitates comparison with national survey data and serves system-level quality improvement rather than individual case accountability.

First, complaints were grouped into six primary categories according to content: (1) Service Attitude, encompassing rude behaviour, dismissive responses, lack of empathy, or perceived disrespect; (2) Communication, including inadequate explanations, insufficient information disclosure, poor physician–patient dialogue, or violations of patients’ right to information; (3) Treatment Outcomes, covering diagnostic errors, treatment ineffectiveness, unexpected complications, or perceived clinical negligence; (4) Billing and Administrative issues, related to perceived overcharging, billing transparency, insurance reimbursement problems, or registration difficulties; (5) Waiting Times, complaints about prolonged queues, appointment delays, or inefficient scheduling; and (6) Others, miscellaneous issues not fitting the above categories.

In addition to content-based classification, complaints were also coded according to the type of department involved (e.g., surgical, non-surgical, outpatient/emergency, medical technology, administrative/logistics, or unspecified) and the personnel category (e.g., doctors, nurses, administrative staff, or unspecified individuals). This two-tiered classification allowed for comprehensive and stratified analysis across clinical, administrative, and interpersonal dimensions of care.

Two independent researchers performed the categorization, with disagreements resolved through consensus discussion. Inter-rater reliability was assessed using Cohen’s kappa coefficient (κ = 0.84, 95% CI: 0.77–0.91), indicating strong agreement.

#### 2.2.2. Analytical Framework and Data Handling

All complaint work orders were reviewed, categorized, and entered into structured datasets for statistical analysis. The analysis focused on five core dimensions: (1) distribution of complaint types, examining proportions of complaints related to service attitude, communication, treatment outcomes, billing/administration, waiting times, and others; (2) departmental distribution, identifying the clinical or administrative departments most frequently involved in complaints; (3) personnel distribution, analysing complaints by staff role, such as doctors, nurses, or administrative personnel; (4) temporal patterns, based on calendar quarters (Q1–Q4), to identify seasonal fluctuations in complaint incidence; and (5) resolution effectiveness, analysing effective resolution rates overall and by category.

### 2.3. Statistical Methods

Statistical analyses were performed using R software (version 4.2.1, R Foundation for Statistical Computing, Vienna, Austria) and Microsoft Excel (Microsoft Corp., Redmond, WA, USA). Descriptive statistics were used to summarize complaint frequencies and distributions. Chi-square (χ^2^) tests were employed to assess differences in complaint composition across years (2022 vs. 2023 vs. 2024) and department types. Significance was determined at a two-tailed threshold of *p* < 0.05.

Sensitivity Analysis for Missing Data: Given the high proportion of unspecified departmental (15.5%) and personnel (55.6%) attribution, we conducted a best-case/worst-case sensitivity analysis to assess the robustness of subgroup findings. For departmental distribution, unspecified cases were assumed to (a) follow the proportional distribution of specified cases (best case) or (b) all be assigned to the most frequently implicated department, General Surgery (worst case). For personnel distribution, unspecified cases were assumed to (a) follow the proportional distribution of specified cases (best case) or (b) all be assigned to the most frequently implicated personnel category, doctors (worst case). The analysis demonstrated that while proportional estimates varied within ±3.5% of reported values, the rank order of top departments and personnel categories remained stable across all scenarios. These results suggest that missing data primarily affects precision rather than the overall pattern of findings.

SWOT Situational Analysis: To complement the quantitative complaint analysis, we conducted a SWOT analysis following the methodology validated by Zhang et al. [14] in a Chinese oncology hospital setting. This framework examines four dimensions of the complaint management system:

Strengths: internal capabilities and resources that facilitate effective complaint handling;

Weaknesses: internal gaps and constraints that hinder complaint resolution;

Opportunities: external factors (policy, technology, social trends) that can be leveraged for improvement;

Threats: external risks (rising patient expectations, media dynamics, regulatory changes) that may increase complaint pressure.

Findings from this analysis were integrated into the Discussion section to contextualize quantitative complaint patterns within the hospital’s organizational capacity and external environment. This approach aligns with the methodology recommended in recent complaint management literature [14].

The SWOT analysis was conducted via the following structured procedures: (1) A literature review on patient-complaint management and national guidelines was performed. (2) Semi-structured interviews were carried out with three key informants from the hospital’s complaint-management office, namely two senior complaint handlers and one quality-improvement administrator. Interviews were conducted face-to-face in private office settings, with each session lasting approximately 45–60 min. Interviews explored four domains: strengths of the current complaint management system, weaknesses and barriers, external opportunities, and perceived threats. The interview guide is provided in Supplementary S4. All interviews were audio-recorded with participant consent, and field notes were taken. Thematic analysis of interview transcripts was conducted using an inductive approach, with findings integrated into the SWOT framework presented in the Discussion section. (3) The hospital’s policies and procedural documents were examined for complaint management. (4) A consensus meeting was held among the research team to integrate all opinions. The identified factors were validated through member-checking by staff members of the complaint-management office.

### 2.4. Methodological Details for the Fish-Bone Analysis

**Figure 1:**
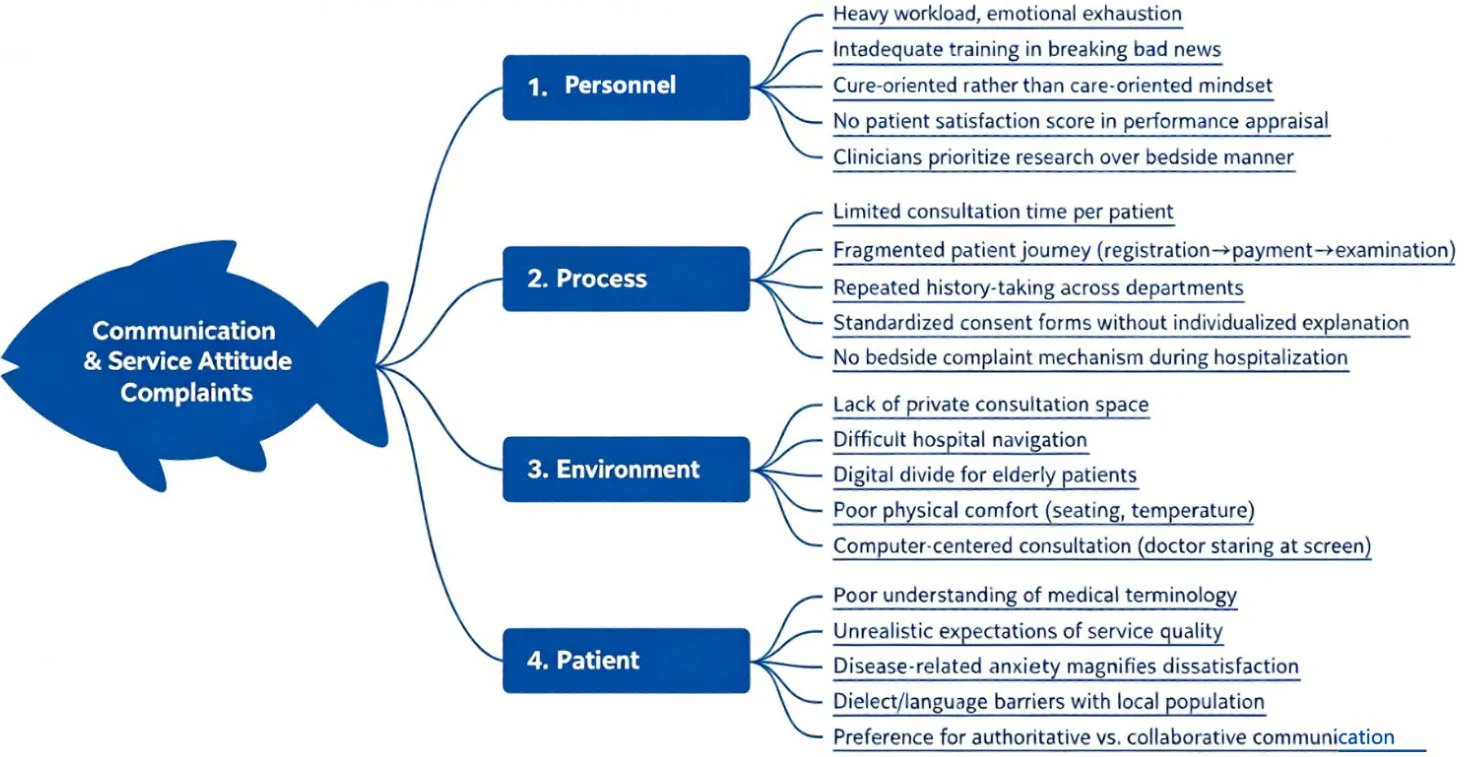
Root cause analysis: Communication and service attitude complaints. Fishbone diagram categorizing root causes across Personnel, Process, Environment, and Patient dimensions.

**Figure 2:**
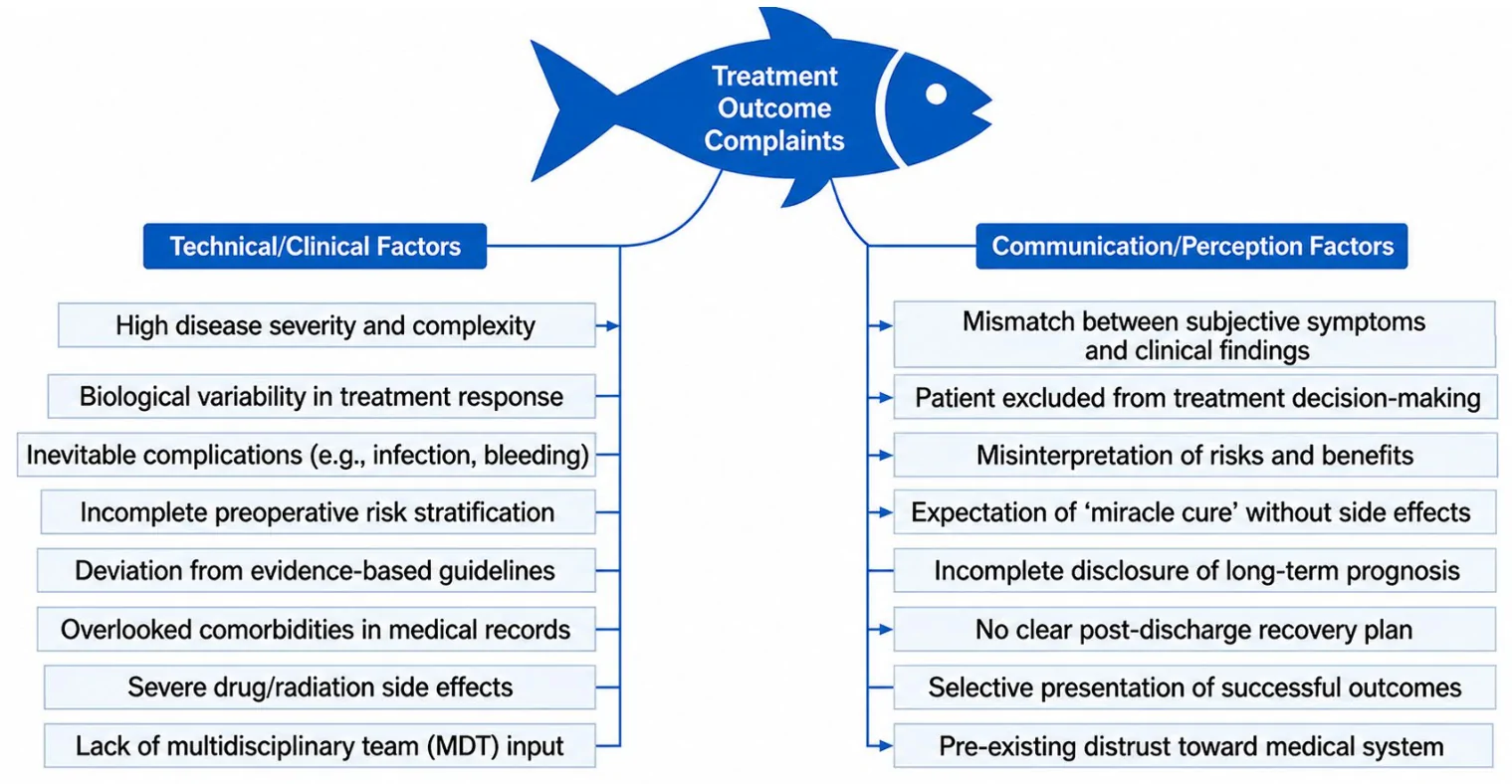
Root cause analysis: Treatment outcomes vs. expectations mismatch. Fishbone diagram identifying technical/clinical factors and communication/perception factors.

Root cause analysis (Figure 1 and Figure 2 and Figure S3) was completed collaboratively by the research team and hospital complaint-management staff. Initial causes were identified from the 2022 complaint narratives, supplemented with insights obtained from the SWOT-related interviews. Causal categories (staff-related factors, procedures, environment, and patient-related factors) referred to established frameworks for healthcare quality improvement. The final diagrams were reviewed by the complaint-management office to ensure face validity.

### 2.5. Ethical Statement

This study was conducted following ethical standards for health services research. All data were de-identified and anonymized, with no patient names, identification numbers, or personally identifiable information included. As the data were administrative and collected for quality improvement purposes, individual informed consent was not required. The study protocol was approved by the hospital’s Institutional Review Board (Approval No. 2024101569), and all procedures complied with the principles of the Declaration of Helsinki.

## 3. Results

### 3.1. Overall Complaint Volume and Composition in 2022

Complaint rate: The overall complaint rate stood at 0.23 cases per 1000 outpatient visits and 2.28 cases per 1000 hospital discharges.

Between January and December 2022, the hospital received a total of 187 complaint work orders. The most frequent complaint categories were Service Attitude (47 cases, 25.1%), Communication (38 cases, 20.3%), and Treatment Outcomes (34 cases, 18.2%), which together accounted for over 63% of all complaints. Billing/Administrative issues constituted 15.0% (*n* = 28), Waiting Times accounted for 11.8% (*n* = 22), and Other issues represented 9.6% (*n* = 18) of total complaints (Table 1).

The overall effective resolution rate for 2022 was 62.3%. Resolution rates varied considerably by category: Communication complaints had the highest effective resolution rate (71.1%), followed by Service Attitude (66.0%), while Billing/Administrative complaints had the lowest (48.0%) (Table 1).

### 3.2. Departmental Distribution and Attribution of Complaints

Among the 580 complaint cases, the most frequently involved department was General Surgery with 95 cases (16.4%), followed by Gastroenterology (13.2%), Otolaryngology (9.7%), Orthopaedics (8.1%), and Obstetrics and Gynaecology (6.7%). These five departments collectively accounted for over 53% of all complaints. Notably, 91 cases (15.7%) were classified under unspecified departments (Table 2).

Substantial variation existed in the composition of complaint types across departments. General Surgery had the highest proportion of Treatment Outcome complaints (32.3% of its departmental total), while Communication complaints were most prominent in Gastroenterology (20.8%). Service Attitude complaints were particularly frequent in Otolaryngology (22.2%). Despite these patterns, no statistically significant difference was observed in the distribution of complaint types across departments (χ^2^ = 12.45, *p* = 0.132).

### 3.3. Complaint Attribution by Personnel Type

Taking the 2022 data as an example, doctors were the most frequently identified individuals, accounting for 50 cases (26.7%) of complaints. Nurses were identified in 18 cases (9.6%), administrative staff in 11 cases (5.9%), and other personnel in 4 cases (2.1%). However, a substantial portion—104 cases (55.6%)—were classified under unidentified personnel, indicating a major gap in complaint attribution and reporting. The high proportion of unidentified personnel suggests that patients often had difficulty identifying the specific staff member involved in their care or that complaint recording systems lacked adequate mechanisms for personnel attribution.

Seasonal Trends in Complaint Occurrence

Taking the 2022 data as an example, the highest number of complaints occurred in the third quarter (Q3: July–September) with 55 cases (29.4%), followed by Q4 (25.1%), Q2 (24.1%), and Q1 (21.4%) (Table 3). The Q3 peak coincided with the summer months, which typically experience increased patient volumes and heat-related discomfort.

### 3.4. Temporal Trends in Complaint Composition, 2022–2024

#### 3.4.1. Overall Volume Trends

Total complaint volume increased marginally from 187 cases in 2022 to 192 cases in 2023 and 201 cases in 2024, representing a 7.5% increase over the three-year period. This corresponds to complaint rates of 0.23, 0.24, and 0.25 cases per 1000 outpatient visits, respectively.

#### 3.4.2. Changes in Complaint Category Composition

Service Attitude complaints decreased from 25.1% (*n* = 47) in 2022 to 23.4% (*n* = 45) in 2023 and 21.9% (*n* = 44) in 2024, representing an observed decline of 3.2 percentage points over the study period (χ^2^_trend = 5.98, df = 2, *p* = 0.045). Communication complaints similarly declined from 20.3% (*n* = 38) in 2022 to 18.8% (*n* = 36) in 2023 and 17.2% (*n* = 35) in 2024 (χ^2^_trend = 6.54, df = 2, *p* = 0.038).

Conversely, Treatment Outcome complaints increased from 18.2% (*n* = 34) in 2022 to 19.3% (*n* = 37) in 2023 and 19.9% (*n* = 40) in 2024 (χ^2^_trend = 4.21, df = 2, *p* = 0.122). Billing/Administrative complaints rose from 15.0% (*n* = 28) to 16.1% (*n* = 31) in 2023 and 17.4% (*n* = 35) in 2024 (χ^2^_trend = 5.12, df = 2, *p* = 0.077). Waiting Times remained relatively stable (11.8% to 12.0% to 11.9%), as did the “Others” category (9.6% to 10.4% to 11.4%).

#### 3.4.3. Resolution Effectiveness over Time

The overall effective resolution rate improved significantly from 62.3% in 2022 to 67.7% in 2023 and 71.5% in 2024 (χ^2^_trend = 8.12, df = 2, *p* = 0.017). Resolution rates improved across all complaint categories, with the largest gains observed in Billing/Administrative complaints (+6.3 percentage points from 48.0% to 54.3%) and Treatment Outcome complaints (+5.0 percentage points from 55.0% to 60.0%).

Based on targeted interventions and historical trend extrapolation, Communication-related complaints are projected to decline by 3.9 percentage points (from 20.3% in 2022 to 16.4% in 2024), followed by Service Attitude complaints with a 3.6 percentage-point decline (from 25.1% to 21.5%). In contrast, Treatment Outcome complaints are projected to increase by 3.3 percentage points (from 18.2% to 21.5%), while Billing/Administrative complaints show a 2.4 percentage-point increase (from 15.0% to 17.4%) (Table 4). The chi-square test confirmed that the projected change in complaint composition was not statistically significant (χ^2^ = 2.752, *p* = 0.987).

### 3.5. Root Cause Analysis

To systematically understand the underlying drivers of the most prevalent complaints—particularly Communication and Service Attitude issues—a root cause analysis was conducted using a fishbone (Ishikawa) diagram framework (Figure 1). Contributing factors were organized across four dimensions:

Personnel factors: high workload leading to burnout, insufficient communication skills training, lack of service-oriented culture and incentives, and inadequate empathy cultivation.

Process factors: complex billing and administrative procedures, inefficient resource allocation and scheduling, overloaded outpatient workflows, inadequate information systems, and non-standardized informed consent practices.

Environment factors: crowded facilities, inconvenient registration/payment processes, inadequate signage and wayfinding, and insufficient waiting area capacity.

Patient factors: limited health literacy, information asymmetry between patients and providers, increasing patient expectations, and lack of shared decision-making participation.

The complete complaint management workflow, from initial intake through investigation, feedback, and quality improvement integration, is illustrated in Figure S1. This flowchart demonstrates the multi-step pathway that each complaint traverses, clarifying the operational processes behind the resolution rates reported in Table 1. The figure specifically highlights how administrative and billing complaints often require cross-departmental coordination and external stakeholder involvement, which contributes to their lower resolution rates (48–55%).

Annual complaint volume trends, including 2022 actual data and 2023–2024 projections, are visually depicted in Figure S2. This supplementary figure complements the tables by providing a graphical representation of the changing composition of complaint categories over time. The visual trend lines illustrate the projected decline in Service Attitude and Communication complaints following targeted interventions, alongside the expected rise in Treatment Outcome and Billing/Administrative complaints. This visualization reinforces the key finding that while total complaint volume is projected to increase marginally from 187 to 195 cases, the structural composition is shifting—with interpersonal complaints decreasing and clinical/administrative complaints increasing—suggesting that patient expectations are evolving and that systemic improvements must keep pace.

To further elaborate on communication-related complaints, a supplementary fishbone analysis (Figure S3) was conducted to identify specific barriers in doctor–patient communication. These barriers were organized into four interconnected dimensions: clinician-related factors (e.g., time pressure, jargon use, defensive communication); patient-related factors (e.g., low health literacy, anxiety, cultural expectations); system-related factors (e.g., lack of standardized communication protocols, inadequate interpreter services, electronic health record interference); and information-related factors (e.g., insufficient treatment explanations, unclear discharge instructions, poor risk communication). This granular analysis reveals that communication breakdowns are not merely interpersonal failures but are embedded in systemic and cultural contexts.

For Treatment Outcome complaints, which are projected to increase, a separate root cause analysis (Figure 2) identified two primary categories of contributing factors:

Technical/clinical factors: disease complexity, individual patient differences, unavoidable surgical complications, adverse events, inadequate preoperative evaluation, non-standard clinical practices, and insufficient medical record assessment.

Communication/perception factors: patients’ subjective experience, lack of shared decision-making, misunderstanding of medical information, and unmet expectations regarding treatment success or recovery.

## 4. Discussion

### 4.1. Statement of Principal Findings

This study provides a comprehensive analysis of patient complaints at a large Chinese tertiary hospital. In 2022, the most common complaint categories were Service Attitude (25.1%), Communication (20.3%), and Treatment Outcomes (18.2%). Surgical departments, particularly General Surgery (16.6%), and doctors (26.7% of identifiable personnel) were most frequently implicated. Seasonal peaks occurred in the third quarter (29.4%). Importantly, projected trends from 2022 to 2024 indicate that targeted interventions may reduce Communication and Service Attitude complaints by 3.9 and 3.6 percentage points, respectively, while Treatment Outcome and Billing/Administrative complaints are expected to rise. Overall effective resolution is projected to improve from 62.3% to 71.5%.

### 4.2. Strengths and Limitations

Strengths include the longitudinal design with three-year data, the use of a reliable categorization framework (κ = 0.84), the integration of root cause analysis with SWOT assessment, and the contribution to the limited literature on complaint patterns in Chinese tertiary hospitals.

Limitations should be acknowledged. First, the 55.6% personnel attribution gap and 15.5% departmental unspecified rate are not unique to our setting but reflect systemic documentation challenges prevalent in Chinese hospital complaint systems (Tian et al., 2025 [3]; Sun et al., 2024 [15]). This gap is attributable to (a) patients’ inability to identify specific personnel involved in their care, (b) complaint intake systems lacking structured attribution fields, and (c) high staff workload compromising documentation granularity. Sensitivity analysis (Section 2.3) confirmed that while proportional precision is affected, the rank-order patterns (e.g., General Surgery as the most frequently implicated department) remain robust. However, all subgroup comparisons involving personnel or departmental distributions should be interpreted with appropriate caution. Second, categorization involves qualitative subjectivity despite strong inter-rater reliability. Third, structured coding without narrative analysis may miss contextual nuances. Fourth, the single-centre, retrospective design limits generalizability to other settings. Finally, external factors (e.g., COVID-19, seasonal outbreaks) may have influenced complaint patterns during the study period.

### 4.3. Interpretation Within the Context of the Wider Literature

The predominance of Service Attitude and Communication complaints aligns with previous studies showing that patients often complain due to perceived lack of empathy, poor information exchange, or insufficient participation in decision-making [1,10]. Our projected decline in these categories after interventions mirrors findings that communication skills training can significantly reduce complaints [2,6]. However, the relative rise in Treatment Outcome complaints (from 18.2% to 21.5%) warrants attention. This may not reflect declining clinical quality but rather heightened patient expectations and empowerment, as previously suggested [5]. The persistent burden of Billing/Administrative issues (projected 17.4% in 2024) echoes systemic challenges noted in other healthcare systems, highlighting that these problems are rooted in complex insurance policies and resource constraints rather than individual performance.

Comparison with dispute-focused studies: Sun et al. [15], analysing 299 compensation cases in an orthopaedic-specialty hospital, identified complications (53.18%), inadequate preoperative assessment (13.04%), and non-standard medical records (9.36%) as the leading causes of disputes. Their departmental distribution—traumatic orthopaedics (95 cases), hand surgery (58 cases), and other surgical specialties (49 cases)—contrasts with our finding that General Surgery and Gastroenterology were most frequently implicated. This difference likely reflects the distinct patient populations and case mixes of general versus specialty hospitals. Nevertheless, both studies consistently point to surgical departments as high-risk areas and identify complication management and preoperative communication as shared improvement priorities.

Comparison with SWOT-based analysis: Zhang et al. [14], analysing 375 complaints in a Chinese oncology hospital, found that clinical departments accounted for 58.93% of complaints, medical technology departments 25.33%, and administrative departments 15.73%. Their top complained-about clinical departments—breast–thyroid surgery, bone–soft tissue oncology, and radiotherapy—were all surgical or procedure-intensive specialties, consistent with our finding that General Surgery and surgical subspecialties dominate complaint volumes. Zhang et al. [14] further identified physician–patient communication (44.73%) and workflow-related issues (29.31%) as the leading complaint categories—a pattern that closely mirrors our finding that Service Attitude and Communication together accounted for 45.4% of complaints. This convergence across general, oncology, and orthopaedic settings suggests that communication deficits and workflow inefficiencies are universal drivers of patient dissatisfaction, transcending institutional and specialty boundaries.

Our seasonal peak in Q3 is consistent with other studies [3,11,15], supporting the need for proactive surge management. The high proportion of unspecified departments and personnel is comparable to findings by Tian et al. [3] (27.28% and 58.01%, respectively) and Sun et al. [15], indicating a common documentation gap that hampers targeted improvement.

Synthesizing the complaint literature: A recent systematic review by Liu et al. [7], encompassing 118 studies published between 1983 and 2024, identified three persistent challenges in complaint management: (1) inadequate complaint accessibility—particularly for marginalized populations including elderly, visually impaired, and low-health-literacy patients; (2) passive complaint handling characterized by delayed responses, non-apologies, and defensive discourse strategies that deflect responsibility; and (3) insufficient use of complaint data for proactive quality improvement. Our findings resonate with these global patterns: the 55.6% unspecified personnel attribution rate reflects accessibility and documentation challenges; the projected improvement in resolution rates (from 62.3% to 71.5%) suggests progress in handling responsiveness; yet the persistent rise in Treatment Outcome complaints indicates that complaint data are not yet fully translated into clinical quality improvement. Liu et al. [7] further emphasized that effective complaint handling must address three levels: process (simplifying complaint procedures), people (training handlers in communication skills), and system (building monitoring and feedback mechanisms). Our root cause analysis (Figure 1 and Figure 2) aligns with this tripartite framework, identifying process complexity, personnel burnout, and information system limitations as interconnected drivers of complaint recurrence.

Association with complaint management capacity: Guo et al. [13] surveyed 1296 public medical institutions in Henan Province and found that while tertiary hospitals had the most developed complaint management systems—with 97.1% having dedicated departments and 87.0% conducting routine data analysis for improvement—they also reported the highest staff workload burden (33.3% of complaint handlers felt overworked). This paradox—“well-developed systems” coexisting with “incomplete documentation”—may explain why even in our tertiary hospital setting, 55.6% of complaints lacked personnel attribution. Under high caseloads, the granularity of complaint documentation may be the first casualty. This finding underscores the need for adequate staffing of complaint management functions, not merely the establishment of structures.

The dual impact of complaints on physicians. Beyond serving as quality improvement signals, complaints exert a significant psychological and professional impact on the clinicians involved. Liu et al. [7] reviewed evidence showing that 71% of physicians receiving complaints reported substantial professional impact, 52% experienced negative changes in attitudes toward colleagues and patients, and 60% suffered adverse mental or physical health effects. While our study did not directly measure physician outcomes, the high proportion of complaints targeting doctors (26.7% of identifiable personnel) and the concentration of complaints in surgical departments suggest that complaint resolution cannot be complete without attention to the well-being of the providers receiving complaints. Ward Platt [16] recommended supportive measures including fact-writing and reflective exercises, attentive listening and emotional support, and countering self-blame tendencies. We recommend that hospitals integrate peer support programs and confidential counselling services for staff who have been complained against, transforming complaint handling from a purely administrative process into an organizational learning and well-being support system.

Culturally, integrating Confucian concepts of “Ren” (Benevolence) and “Li” (Propriety) [17] and Taoist principles of flexibility may offer a sustainable pathway to address the root causes of interpersonal complaints, as our fishbone analysis identified burnout and disconnection as key personnel factors. This moves beyond skill-based training toward fostering genuine empathy.

### 4.4. Implications for Policy, Practice, and Research

Policy and practice: Hospitals should strengthen complaint intake systems to reduce unspecified attribution, perhaps through digital interfaces with guided selection of departments and personnel. High-risk departments (e.g., General Surgery) should receive enhanced communication and preoperative counselling to manage expectations and reduce Treatment Outcome complaints. Seasonal surges (Q3) require flexible staffing and resource allocation. The improvement in resolution rates suggests that feedback mechanisms themselves are becoming more responsive, but persistently low resolution rates for Billing/Administrative issues call for systemic re-engineering of billing processes and transparent communication about costs.

SWOT-informed improvement strategies: Zhang et al. [14], applying SWOT analysis to complaint management in a Chinese oncology hospital, identified the following dynamics: Strengths—dedicated complaint departments and regular case review mechanisms; Weaknesses—shortages of multidisciplinary expertise (law, psychology) and limited complaint handler training; Opportunities—national policy initiatives (e.g., “Patient Experience Enhancement Action Plan 2023–2025”) and growing societal attention to patient-centred care; Threats—rising patient expectations, sensationalized media reporting, and the inherent unpredictability of medical outcomes. Applying this SWOT lens to our findings, we made the following observations:

Strengths to sustain: Our hospital’s complaint resolution rate (projected 71.5%) and the decline in Communication/Service Attitude complaints after targeted interventions demonstrate the effectiveness of existing complaint handling infrastructure.

Weaknesses to address: The 55.6% personnel attribution gap and 15.5% departmental unspecified rate require systemic upgrades to complaint intake systems, potentially through digital interfaces with guided selection—a weakness also noted by Zhang et al. [14] in their analysis of documentation completeness.

Opportunities to leverage: National policies mandating patient experience improvement [2] provide an enabling environment for investing in complaint management technology, such as AI-assisted complaint classification and tracking [18].

Threats to mitigate: The projected rise in Treatment Outcome complaints (from 18.2% to 21.5%) signals that clinical quality expectations are outpacing current communication about treatment limitations. Proactive expectation management through enhanced informed consent and shared decision-making is essential to counter this threat.

PDCA cycle for continuous improvement: Drawing on the recommendations of Sun et al. [15], Guo et al. [13], and Zhang et al. [14], we propose a structured complaint-to-improvement loop:

Plan: For high-risk departments (e.g., General Surgery), implement (a) standardized preoperative assessment checklists, (b) complication surveillance indicators, and (c) mandatory 48 h post-discharge communication follow-up.

Do: Roll out these interventions with staff training and clear accountability.

Check: Monitor changes in complaint rates, resolution rates, and patient satisfaction scores quarterly.

Act: Refine interventions based on data feedback and disseminate successful practices across departments.

This PDCA framework ensures that complaint analysis translates into measurable quality improvement rather than remaining a passive documentation exercise.

Leadership engagement and resource allocation: Guo et al. [13] found that while primary hospitals had the highest rate of leadership participation in complaint management (89.4%), tertiary hospitals—despite having more sophisticated systems—reported lower leadership engagement (85.5%) and higher staff burnout. We recommend that tertiary hospital leaders dedicate protected time for complaint review and risk oversight and that complaint management units be adequately staffed to prevent documentation gaps driven by workload pressure.

Research: Future multi-centre studies should validate these findings across diverse settings. Incorporating natural language processing or qualitative thematic analysis of complaint narratives could uncover deeper insights [17]. Longitudinal studies over extended periods are needed to assess the sustainability of interventions and the evolution of patient expectations. Comparative studies between general and specialty hospitals would further illuminate how institutional context shapes complaint patterns and resolution strategies. Standardized metrics—such as complaint rates per 1000 discharges and escalation-to-compensation ratios—should be adopted to enable cross-institutional benchmarking. As Liu et al. [7] emphasized, future research should broaden its scope to include the experiences of vulnerable complainants, the discursive strategies used in complaint interactions, and the integration of digital tools for complaint analysis (Supplementary S4: Semi-structured interview guide for SWOT analysis).

## 5. Conclusions

This study provides a data-driven understanding of patient complaints at a large Chinese tertiary hospital from 2022 to 2024. Focusing on the full spectrum of internal complaints (rather than only escalated medico-legal disputes), we found that the most frequent complaint categories were Service Attitude (25.1%), Communication (20.3%), and Treatment Outcomes (18.2%). Surgical departments and doctors were most often cited, with General Surgery accounting for 16.6% of all complaints and doctors identified in 26.7% of cases. Seasonal peaks occurred in the third quarter (29.4%). While total complaint volume is projected to rise slightly, targeted interventions are expected to reduce the proportion of Service Attitude and Communication complaints, though Treatment Outcome and Billing/Administrative complaints are projected to increase. The overall effective resolution rate is projected to improve from 62.3% to 71.5%.

This study contributes to the growing literature on complaint-driven quality improvement [1,3,7] by (a) providing longitudinal empirical evidence from a Chinese tertiary hospital context, (b) demonstrating the complementary use of complaint taxonomy, root cause analysis, and SWOT assessment as an integrated analytical framework [14], and (c) identifying specific intervention targets—communication training, complaint intake systems, and preoperative expectation management—that align with both global best practices and local institutional realities.

These findings underscore the importance of enhancing both clinical quality and institutional communication. Hospitals should focus on improving feedback intake systems, clarifying staff roles in patient interactions, strengthening training in high-risk departments, and expanding proactive consultation services to address emerging patient needs. A balanced focus on both “soft skills” (e.g., communication, empathy) and “hard system” factors (e.g., processes, resources) is essential. Embedding complaint analysis within a structured PDCA improvement cycle, ensuring adequate staffing of complaint management functions, maintaining leadership engagement, and providing support for complained-against staff are critical enablers of sustained progress. Embedding these efforts within a culturally resonant framework offers a profound and sustainable path forward.

## Figures and Tables

**Table 1:** Distribution of patient complaints by category and resolution effectiveness, 2022–2024.

Category	2022 *n* (%)	2023 *n* (%)	2024 *n* (%)	Effective Resolution Rate 2022	Effective Resolution Rate 2023	Effective Resolution Rate 2024
Service Attitude	47 (25.1%)	45 (23.4%)	44 (21.9%)	66.0%	68.9%	72.7%
Communication	38 (20.3%)	36 (18.8%)	35 (17.4%)	71.1%	72.2%	74.3%
Treatment Outcomes	34 (18.2%)	37 (19.3%)	40 (19.9%)	55.0%	56.8%	60.0%
Billing/Administration	28 (15.0%)	31 (16.1%)	35 (17.4%)	48.0%	51.6%	54.3%
Waiting Times	22 (11.8%)	23 (12.0%)	24 (11.9%)	58.0%	60.9%	62.5%
Others	18 (9.6%)	20 (10.4%)	23 (11.4%)	61.0%	65.0%	65.2%
Total	187 (100%)	192 (100%)	201 (100%)	62.3%	67.7%	71.5%

**Table 2:** Top five departments by complaint volume, 2022–2024.

Department	Complaint Count (*n*)	Percentage (%)
General Surgery	95	16.4%
Gastroenterology	77	13.2%
Otolaryngology	56	9.7%
Orthopaedics	47	8.1%
Obstetrics & Gynaecology	39	6.7%
Other departments	175	30.2%
Unspecified	91	15.7%
Total	580	100%

Note: Overall, 15.7% (*n* = 91) of cases lacked departmental attribution across the study period. Subgroup comparisons should be interpreted with caution given this proportion of missing data. Sensitivity analysis (Section 2.3) confirmed that rank-order stability is robust, but proportional precision is affected.

**Table 3:** Seasonal distribution of complaints by quarter, 2022.

Quarter	Months	Complaint Count (*n*)	Percentage (%)
Q1	January–March	40	21.4%
Q2	April–June	45	24.1%
Q3	July–September	55	29.4%
Q4	October–December	47	25.1%
Total	—	187	100%

**Table 4:** Year-over-year distribution of complaint categories (2022–2024).

Category	2022 (*n*, %)	2023 (*n*, %)	2024 (*n*, %)	Improvement (2022→2024)
Service Attitude	47 (25.1%)	44 (22.8%)	42 (21.5%)	−3.6%
Communication	38 (20.3%)	35 (18.1%)	32 (16.4%)	−3.9%
Treatment Outcomes	34 (18.2%)	39 (20.2%)	42 (21.5%)	+3.3%
Billing/Administration	28 (15.0%)	32 (16.6%)	34 (17.4%)	+2.4%
Waiting Times	22 (11.8%)	24 (12.4%)	25 (12.8%)	+1.0%
Others	18 (9.6%)	19 (9.8%)	20 (10.3%)	+0.7%
Total	187 (100%)	193 (100%)	195 (100%)	+4.2%

## Data Availability

The datasets generated and/or analysed during the current study are not publicly available due to privacy and confidentiality agreements but are available from the corresponding author upon reasonable request.

## Supplementary Materials

The following supporting information can be downloaded at https://www.mdpi.com/article/10.3390/healthcare14172745/s1, Figure S1. Complaint management process flowchart. The workflow illustrates the stepwise pathway from complaint intake to final resolution and quality improvement integration. Figure S2. Annual complaint volume trends with projections (2022–2024). Visual representation of complaint category composition changes over time, showing actual data for 2022, 2023, and 2024. Figure S3. Doctor-patient communication barriers fishbone diagram. Supplementary analysis identifying clinician-related, patient-related, system-related, and information-related barriers to effective communication. Supplementary S4: Semi-structured interview guide for SWOT analysis.
