# Supplementary material for: Characteristics of Patient Complaints in a Large Tertiary Hospital in China: A Longitudinal Analysis from 2022 to 2024
Source: Healthcare (Basel). 2026 Aug 28;14(17):2745. doi: 10.3390/healthcare14172745 (PMC13565842; doi:10.3390/healthcare14172745)
Supplement: Supplementary file 1 [file healthcare-14-02745-s001.zip › healthcare-4462329-supplementary.pdf]

SUPPLEMENTARY FILES

Figure S1. Complaint management process flowchart. The workflow illustrates the stepwise pathway from complaint intake to final resolution and quality improvement integration.

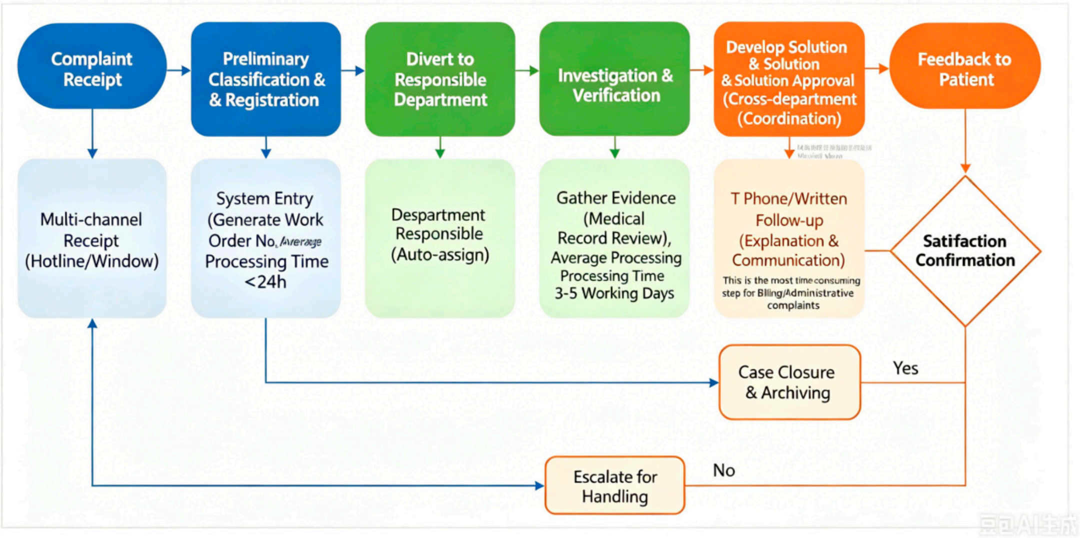

Figure S2. Annual complaint volume trends with projections (2022－2024). Visual representation of complaint category composition changes over time, showing actual data for 2022, 2023, and 2024.

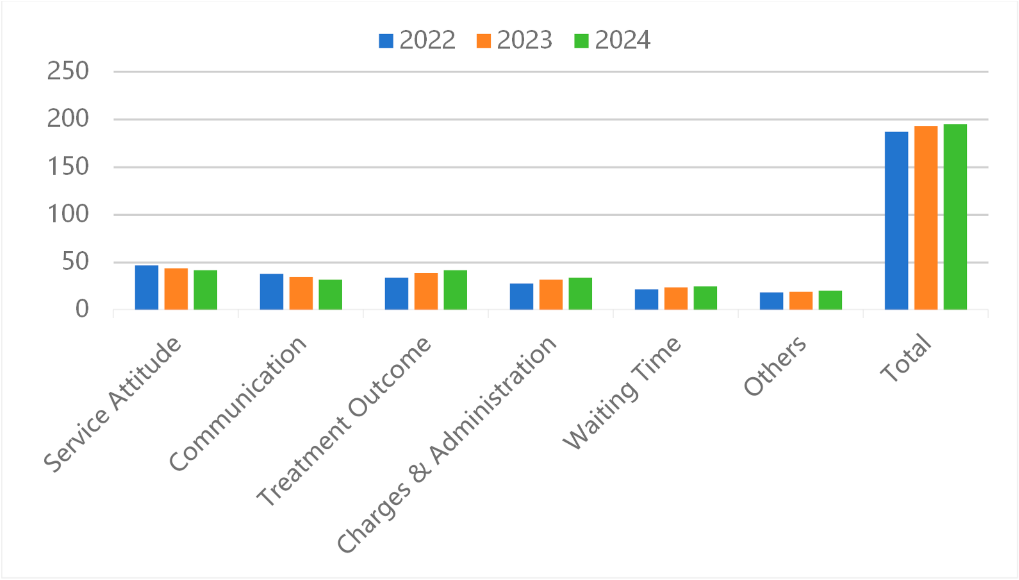

Figure S3. Doctor-patient communication barriers fishbone diagram. Supplementary analysis identifying clinician-related, patient-related, system-related, and information-related barriers to effective communication.

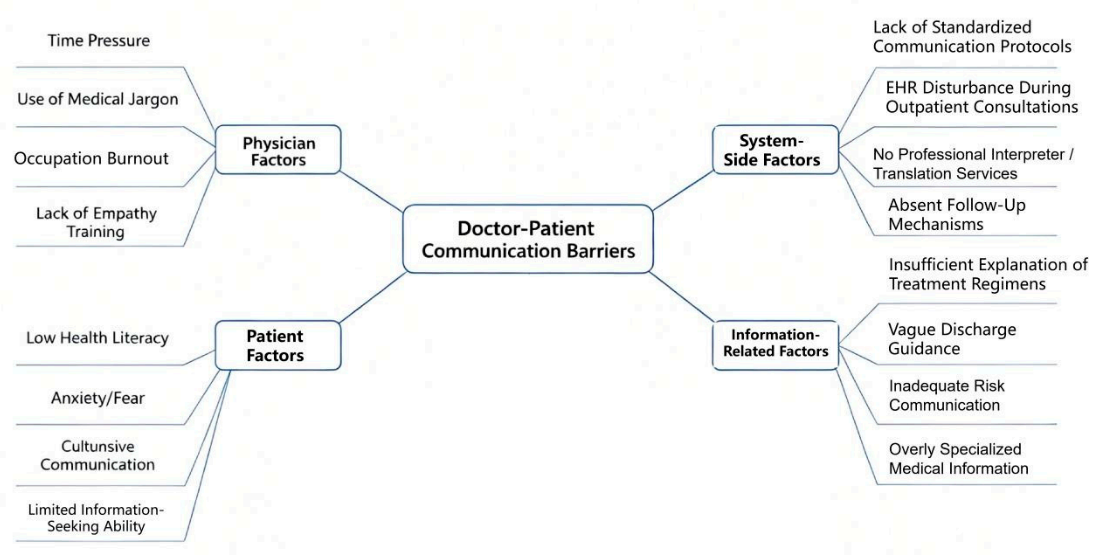

Supplementary S4: Semi-Structured Interview Guide for SWOT Analysis

Introduction: Thank you for participating in this interview. The purpose of this interview is to understand the strengths, weaknesses, opportunities, and threats of the hospital's current complaint management system. Your responses will be used to inform the SWOT analysis in our research. The interview will take approximately 45–60 minutes. With your permission, we will record the session for accurate transcription. All responses will be anonymized and kept confidential.

Domain 1: Strengths

1. What do you consider to be the main strengths of our hospital's current complaint management system?
2. What aspects of the complaint handling process work well from your perspective?
3. What resources (staff, technology, training) support effective complaint resolution?

Domain 2: Weaknesses

1. What are the main challenges or barriers you face in handling patient complaints?
2. What gaps exist in the current complaint intake, documentation, or resolution processes?
3. What factors contribute to low resolution rates for certain complaint categories (e.g., billing)?

Domain 3: Opportunities

1. What external policies, technologies, or social trends could be leveraged to improve complaint management?
2. What opportunities exist for training staff in communication and service attitude?
3. How could digital tools (e.g., AI, NLP) improve complaint analysis?

Domain 4: Threats

1. What external factors increase the risk of complaints (e.g., rising patient expectations, media scrutiny)?

2. What changes in the healthcare environment (policy, regulation, societal attitudes) pose challenges?

3. What are the potential consequences if complaint management does not improve?

Closing: Is there anything else you would like to add about the complaint management system that we have not discussed? Thank you for your time and insights.
